# Efficacy and outcomes of ramucirumab and docetaxel in patients with metastatic non-small cell lung cancer after disease progression on immune checkpoint inhibitor therapy: Results of a monocentric, retrospective analysis

**DOI:** 10.3389/fonc.2023.1012783

**Published:** 2023-03-21

**Authors:** Samuel A. Kareff, Kunal Gawri, Khadeja Khan, Deukwoo Kwon, Estelamari Rodriguez, Gilberto de Lima Lopes, Richa Dawar

**Affiliations:** ^1^ Department of Graduate Medical Education, University of Miami Sylvester Comprehensive Cancer Center/Jackson Memorial Hospital, Miami, FL, United States; ^2^ Department of Medicine, State University of New York-Buffalo, Buffalo, NY, United States; ^3^ Department of Undergraduate Medical Education, University of Miami Miller School of Medicine, Miami, FL, United States; ^4^ Department of Population Health Science and Policy, Icahn School of Medicine at Mount Sinai, New York, NY, United States; ^5^ Department of Medical Oncology, University of Miami Sylvester Comprehensive Cancer Center, Miami, FL, United States

**Keywords:** ramucirumab, docetaxel, platinum-based treatment resistance, metastatic NSCLC, REVEL

## Abstract

Current first-line standard therapy for metastatic non-small cell lung cancer without driver mutations involves chemotherapy and immunotherapy combination. Prior to the advent of immune checkpoint inhibition, REVEL, a randomized phase III trial demonstrated improved progression-free and overall survival with ramucirumab and docetaxel (ram+doc) in patients who failed platinum-based first-line therapy. Long-term outcomes related to second-line ramucirumab and docetaxel after first-line immunotherapy exposure remain unknown. We analyzed outcomes for 35 patients from our center whom received ramucirumab and docetaxel following disease progression on chemotherapy and immunotherapy combination. Median progression-free survival among patients who received ram+doc after exposure to immunotherapy was 6.6 months (95% CI = 5.5 to 14.9 months; *p*<0.0001), and median overall survival was 20.9 months (95% CI = 13.4 months to infinity; *p*<0.0001). These outcomes suggest that there may a synergistic benefit to combining chemotherapy with anti-angiogenic therapy after immunotherapy exposure. Future analyses should be evaluated prospectively and among a larger patient subset.

## Introduction

Non-small cell lung cancer (NSCLC) is the most common type of lung cancer, accounting for nearly 80% of all cases, and often presents in the locally advanced or metastatic settings (1). Currently, a combination of chemotherapy and/or immunotherapy (IO) is considered standard first-line treatment for metastatic NSCLC (mNSCLC) without driver mutations, often tailored based on a patient’s programmed death-ligand 1 (PD-L1) status (2). Before the advent of IO as first-line therapy, REVEL, a randomized, multicenter, phase III clinical trial, demonstrated improved progression-free survival (PFS), overall survival (OS), and quality of life (QoL) with ramucirumab and docetaxel (ram+doc) chemotherapy and antiangiogenic combination in patients whose disease progressed after platinum-based doublet first-line chemotherapy compared with docetaxel alone (3, 4). This combination was proposed utilizing ramucirumab as a complete human monoclonal IgG1 antibody with direct vascular endothelial growth factor receptor 2 (VEGFR2) antagonism given already known improved outcomes with docetaxel in platinum-resistant disease (5). Indeed, this biological rationale of overcoming the demonstrated immunosuppressive effect of VEGF has been proven in other lines of therapy and in combination with immunotherapy, such as the IMPOWER150 study which demonstrated improved outcomes for nonsquamous mNSCLC when combining atezolizumab, bevacizumab, and platinum-doublet chemotherapy in the first-line setting (6). There have been studies that have shown promising results in other forms of platinum-resistant tumor histologies, namely, urothelial and gastric cancers (7, 8). There exist data mostly limited to retrospective cohort analyses in East Asia and Europe discussing responses to ram+doc treatment in patients pretreated with IO-based therapy; however, any synergistic benefit has not been proven for patients with mNSCLC (9). Therefore, our study aims to clarify the efficacy and outcomes of this combined therapeutic approach in patients with paclitaxel-resistant mNSCLC.

## Materials and methods

We performed a retrospective cohort study among all patients with mNSCLC treated at the University of Miami Sylvester Comprehensive Cancer Center. We retrospectively identified all patients with mNSCLC whose disease demonstrated progression after IO-based therapy and then received ram+doc as a subsequent line of therapy between January 1^st^, 2010 and March 1^st^, 2020. A total of thirty-nine patients were identified whom met these inclusion criteria. We subsequently excluded 4 patients with EGFR or ALK driver mutations from our analysis. As such, thirty-five (*n* = 35) patients were included in our final analysis. We assessed patients’ PFS and OS after ram+doc treatment utilizing the Kaplan-Meier method as primary outcomes. We compared our center’s retrospective data with those from REVEL data utilizing a simulation study *via* Wilcoxon test. Since REVEL data was not available for reproduction, we used median PFS and median OS and corresponding 95% CIs to estimate the distribution of median survival time for our dataset compared to that of REVEL using an approximate Bayesian computation (ABC) approach. We also collected information on adverse events (AEs) during ram+doc treatment as a secondary outcome. This study was approved by the University of Miami Institutional Review Board eProst #20170427.

## Results

### Patient characteristics

Of a total 44 patients treated with ramucirumab and docetaxel at our center, we excluded 6 patients with *EGFR* mutation, 1 with *ALK* mutation, and 2 without previous exposure to IO. We report the patient demographics as well as some treatment characteristics for the 35 included patients in **Table 1**. Patients’ age ranged from 45 to 76 years (median 65 years). There were a total 17 females (48.6%) and 18 males (51.4%) represented in the sample. 19 patients identified as Hispanic/Latinx (54.3%), 12 as white (34.3%), and 1 as African American (2.8%). 28 were tobacco users (80%), while 7 were never-tobacco users (20%). All 35 patients received ICI as first-, second-, or third-line therapy for mNSCLC, which was followed immediately by ram+doc upon disease progression. 33 patients’ tumor histology was adenocarcinoma (94.3%), while 2 patients’ tumor histology was squamous cell carcinoma (5.7%). All patients had disease with at least 3 metastatic sites, listed in the following order of frequency: 1) bone, 2) liver, and 3) brain.

**Table 1:** Demographics of patients whom received ramucirumab and docetaxel, and characteristics of treatment, at the University of Miami.

Demographic/Treatment Category	Sub-Category	*n* (%)
Race/Ethnicity	Black	1 (2.9)
	Hispanic/Latinx	19 (54.3)
	White	12 (34.3)
	Other/Multiple	3 (8.6)
Gender	Female	17 (48.6)
	Male	18 (51.4)
Age	40-49	1 (2.9)
	50-59	7 (20)
	60-69	18 (51.4)
	70-79	9 (25.7)
History of Tobacco Use	Yes	28 (80)
	No	7 (20)
Histology	Adenocarcinoma Squamous Cell Carcinoma	33 (94.3) 2 (5.7)
PDL1 Percentage	0% <50% >50% Unknown	14 (40) 7 (20) 5 (14.3) 9 (25.7)
Number of Metastatic Sites	<3 >3	1 (2.9) 34 (97.1)
Sites of Metastases	Bone Liver Brain	24 (68.9) 11 (31.4) 7 (20)
First Line of Treatment	Platinum-doublet + IO Platinum-doublet chemo Platinum-doublet + anti-VEGF IO alone Other chemo	20 (57.1) 11 (31.4) 2 (5.7) 1 (2.9) 1 (2.9)
Ram+Doc Line of Therapy	Second Third Fourth or Beyond	20 (57.1) 11 (31.4) 4 (11.4)

### Primary and secondary patient outcomes

**Figure 1:**
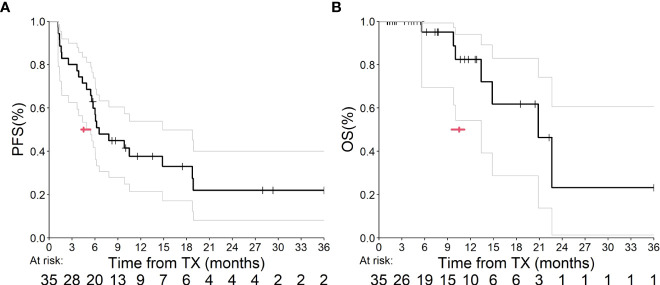
**(A, B)** This graph depicts PFS **(A)** and OS **(B)** from our UM outcomes. The bold line indicates our Kaplan-Meier analysis, and the gray lines represent our 95% CI estimates. The red arrow indicates median estimates within both graphs.

Median PFS (mPFS) among patients whom received ram+doc therapy after IO exposure was 6.6 months (95% confidence interval [CI] = 5.5 to 14.9 months; *p*<0.0001] (**Figure 1A**) and median OS (mOS) was 20.9 months (95% CI = 13.4 months to infinity; *p<*0.0001) (**Figure 1B**). There were no statistically significant differences detected among tumor histology. Since the REVEL data were not available for independent reproduction, we utilized the ABC approach to estimate how many *p*-values are less than 0.0001 in 1,000 tests for simulated two datasets; this approach has been validated elsewhere (10). Given that all 1,000 p-values were less than 0.0001, we found these results to be statistically significant in estimating our CIs. Moreover, since the 95% CIs of our cohort versus that of REVEL did not overlap, we considered these improved mPFS and mOS outcomes to be statistically significant as well.

We observed six patients with treatment-related adverse events related to ramucirumab: two patients with Grade 1 hypertension; two patients with proteinuria (one Grade 1 and one Grade 2); one patient with Grade 3 hemoptysis, and one with Grade 2 fatigue. Three of these patients experiencing adverse events (*n* = 3/7; 42.8%) required dose reduction. Docetaxel use led to 31 adverse events: 15 patients with fatigue, with at least one Grade 3, three patients with skin/nail changes, one patient with Grade 3 myalgias, 2 patients with neutropenia (1 Grade 3), 4 patients with anemia, 4 patients with neuropathy (1 Grade 3), and 2 patients with cough/COPD exacerbation. Three of the patients with Grade 3 adverse events (*n* = 3/4; 75%) required dose reduction of docetaxel. An additional patient demonstrated Grade 2 arthritis that was attributed to previous IO exposure, and it is unclear how ram+doc subsequent therapy mediated this toxicity. In total, seven patients (*n* = 7/35; 20%) required treatment discontinuation during the course of therapy. Further detailed results are available in **Table 2**.

**Table 2:** Treatment-related adverse events related to ramucirumab and docetaxel among patients in the UM Cohort.

Toxicity Variable	All Grades (%)	Grade 3+ (%)
Hypertension (Ram)	2 (5.7)	–
Proteinuria (Ram)	2 (5.7)	–
Bleeding (Ram)	1 (2.9)	1 (2.9)
Fatigue (Ram)	1 (2.9)	–
*Dose-reduction of Ram*	1 (2.9)	1 (2.9)
Fatigue (Doc)	15 (42.9)	1 (2.9)
Skin/nail changes (Doc)	3 (8.6)	–
Myalgias (Doc)	1 (2.9)	1 (2.9)
Neutropenia (Doc)	2 (5.7)	1 (2.9)
Anemia (Doc)	4 (11.4)	–
Neuropathy (Doc)	4 (11.4)	1 (2.9)
Cough/COPD Exacerbation (Doc)	2 (5.7)	–
*Dose-reduction of Doc*	2 (5.7)	2 (5.7)
*Dose-reduction of Ram + Doc*	2 (5.7)	2 (5.7)
Arthralgia (IO)	1 (2.9)	–

This symbol means no value.

## Discussion

In our retrospective analysis, we found a statistically longer mPFS and mOS for patients with mNSCLC treated with ram+doc after progression on IO at our center compared to the original cohort reported in the REVEL study, which first analyzed this combination chemotherapy and antiangiogenic-therapy regimen in 2014 (**Figures 1A, B**). Our results did not show a difference in these primary outcomes based on tumor histology.

These findings are striking in that they demonstrate a possible synergy between IO pre-treatment and exposure to second-line (or beyond) antiangiogenic-therapy, namely, ramucirumab, in combination with docetaxel. These findings have been echoed in other retrospective cohorts in East Asia. For example, a retrospective review of 99 patients in multiple Japanese centers found a statistically significant mPFS response of 5.9 months in those pre-treated with IO compared to those who did not have IO exposure (2.6 months) (11). These findings were further bolstered in a *post-hoc* sub-group analysis of the original REVEL study in which East Asian patients demonstrated a mPFS of 4.88 months and mOS of 15.4 months (12).

Furthermore, our cohort did not demonstrate unexpected and/or significant adverse events, thereby supporting the safety of ram+doc as combination therapy. This observation also mirrors outcomes in other retrospective cohorts, such as a review of 77 patients in Germany that did not demonstrate unexpected toxicities, i.e., no more than 9.09% febrile neutropenia, fatigue, mucositis, stomatitis, or ileus (13). Similarly, another retrospective analysis in Japan estimated up to 16.7% total (and 11.1% Grade 3 or more) pneumonitis, which is more frequent than the 2.9% of pneumonitis as well as COPD exacerbation observed in our cohort (14).

Our findings also echo activity among a similar combination of docetaxel with nintedanib, a tyrosine kinase inhibitor that has activity against multiple kinases including VEGF. This combination was originally approved based on the LUME-Lung1 study which demonstrated improved PFS and OS compared to docetaxel alone, particularly in adenocarcinoma histology, compared to docetaxel alone in the second-line setting (15). Real-world outcomes mirror those reported at our institution after treatment with chemo- and immunotherapy. Specifically, the VARGADO cohort demonstrated a mPFS of 6.4 months, with a 1-year OS rate of 52% in the third-line setting (16). Furthermore, another German cohort reported a mOS of 8.4 months in adenocarcinoma histology specifically (17).

Two strengths of our study are its location and demographics. Specifically, we report herein the first such retrospective analysis consisting of North American patients, with a majority of patients whom identified ethnically as Hispanic/LatinX. Additionally, our cohort’s outcomes rank among the longest PFS and OS benefits recorded with post-IO ram+doc exposure to date. This result will require additional study with similar ethnic and geographic cohorts.

Our analysis has several limitations. First, this is a single-center, retrospective analysis, and as such these observations should be confirmed in a prospective fashion. The Phase II Lung-Map S1800A study evaluated ramucirumab with pembrolizumab combination therapy compared to standard of care chemotherapy, of which two-thirds of the control arm received ram+doc, and was found to demonstrate an OS benefit (17). *Post-hoc* analyses will be required to understand the true PFS and OS estimates seen in this sub-group, however. Additionally, the TREAT-LUNG observational study reported preliminary data for second- and third-line docetaxel vs. ram+doc in patients previously treated with both platinum-based chemotherapy and IO with a subset of patients demonstrating long-term responses (i.e., plateaus in Kaplan-Meier plots) (18). These findings merit closer attention once presented formally in the literature. The greatest limitation of our study is its small size. For example, a larger Japanese cohort of 1,439 patients utilized a propensity score analysis and did not find a PFS or OS advantage with this treatment strategy (19).

Overall, our institution’s experience with this combination chemo- and antiangiogenic-therapy strategy adds to the data related to ram+doc after IO exposure in mNSCLC. Interpretation should be limited given its retrospective timeframe and single-center patient population.

### Resource identification initiative

Ramucirumab RRID: AB_2911024.

## Data availability statement

The raw data supporting the conclusions of this article will be made available by the authors, without undue reservation.

## Ethics statement

The studies involving human participants were reviewed and approved by University of Miami IRB Eprost 20170427. Written informed consent for participation was not required for this study in accordance with the national legislation and the institutional requirements.

## Author contributions

First authorship: SK. Equal contribution: KG, KK, DK, GL, ER. Senior/last authorship: RD. All authors contributed to the article and approved the submitted version.
